# Clinic continuity of care, clinical outcomes and direct costs for COPD in Sweden: a population based cohort study

**DOI:** 10.1080/20018525.2017.1290193

**Published:** 2017-03-03

**Authors:** Sofia Sveréus, Kjell Larsson, Clas Rehnberg

**Affiliations:** ^a^ Department of Learning, Informatics, Management & Ethics (LIME), Medical Management Centre, Karolinska Institutet, Stockholm, Sweden​​; ^b^ Centre for Health Economics, Informatics and Health Services, Stockholm County Council, Stockholm, Sweden; ^c^ National Institute of Environmental Medicine (IMM), Karolinska Institutet, Stockholm, Sweden

**Keywords:** Chronic obstructive pulmonary disease, COPD, health services research, health economics, organization of care, health policy

## Abstract

**Introduction:** In this study we investigate whether clinic level continuity of care (COC) for individuals with chronic obstructive pulmonary disease (COPD) is associated with better health care outcomes and lower costs in a Swedish setting.

**Methods:** Individuals with COPD (N = 20,187) were identified through ICD-10 codes in all Stockholm County health care registries in 2007–2011 (59% female, 40% in the age group 65–74 years). We followed the individuals prospectively for 365 days after their first outpatient visit in 2012. Individual associations between COC and incidence of any hospitalization or emergency department visit and total costs for health care and pharmaceuticals were quantified by regression analysis, controlling for age, sex, comorbidity and number of visits. Clinic level COC was measured through the Bice–Boxerman COC index, grouped into quintiles.

**Results:** At baseline, 26% of the individuals had been hospitalized at least once and 73% had dispensed at least seven prescription drugs (23% at least 16) in the last year. Patients in the lowest COC quintile (Q1) had higher probabilities of any hospitalization and any emergency department visit compared to those in Q5 (odds ratio 2.17 [95% CI 1.95–2.43] and 2.06 [1.86–2.28], respectively). Patients in Q1 also on average had 58% [95% CI: 52–64] higher costs.

**Conclusion:** The findings show robust associations between clinic level COC and outcomes. These results verify the importance of COC, and suggest that clinic level COC is of relevance to both better outcomes for COPD patients and more efficient use of resources.

## Introduction

Continuity of care (COC) is a complex concept. It entails information exchange, disease management and interpersonal relationships, and correlates with a variety of positive health care outcomes.[1–4] For patients with chronic obstructive pulmonary disease (COPD), COC correlates with lower rates of avoidable hospitalization and emergency department visits, fewer complications, lower costs and lower all-cause mortality.[5–7]

In Sweden, COPD is underdiagnosed and undertreated.[8,9] It has been estimated that 8–10% of the adult population have chronic airflow limitation, fulfilling the spirometric criteria of COPD, but less than one in five of those are aware of their diagnosis.[8,10] The mean age at the time of diagnosis is 66 years. A large share of COPD patients suffers from one or several comorbidities, such as hypertension, heart failure, ischemic heart disease, diabetes or depression.[11]

Studies of COC have used different types of data; either patient surveys, which enable specific information gathering, but are expensive and bias prone, or routinely collected data, which are cheap and readily available, but not specifically designed to measure COC.[12,13]

Despite a comprehensive structure of registries in Sweden, studies of COC using routinely collected data are scarce. This is possibly because Swedish register data do not distinguish between individual caregivers, so COC has to be measured in relation to a certain *clinic*, rather than to a certain *clinician.*

A few decades ago, measurement of COC at clinic level was considered suboptimal.[14] This is starting to change, not least in the light of organizational advances such as clinical management protocols, increased emphasis on timely access to care and facilitated information exchange through electronic medical records.[15] Some previous studies have even found clinic-level COC to be more associated with desirable outcomes than clinician-level COC for multi-chronic patients, indicating that a single clinician could not meet the patients’ entire health care needs.[16]

There are some further features which potentially make clinic-level COC relevant in Sweden. The structure of Swedish primary health care has typically comprised multidisciplinary teams, and the Swedish guidelines on COPD management emphasize inter professional cooperation. This includes continuity of care with a number of professional categories, such as physicians, nurses and physiotherapists.[17] In this context it is relevant to investigate whether the association between high COC and positive clinical outcomes found in clinician-level COC studies also hold when COC is measured at the clinic level.

In the present study we therefore investigate whether clinic-level COC for individuals with COPD is associated with better health care outcomes and lower costs in a Swedish setting.

## Material and methods

### Study subjects

We identified the study subjects from a population of 2.1 million inhabitants in Stockholm County, using a comprehensive anonymized individual level database (the VAL database). The VAL database includes complete data on all types of publicly funded public and private care as well as dispensed prescription drugs. It is held by the County Council, the regional body responsible for health care financing and delivery in Stockholm.

**Figure 1.**
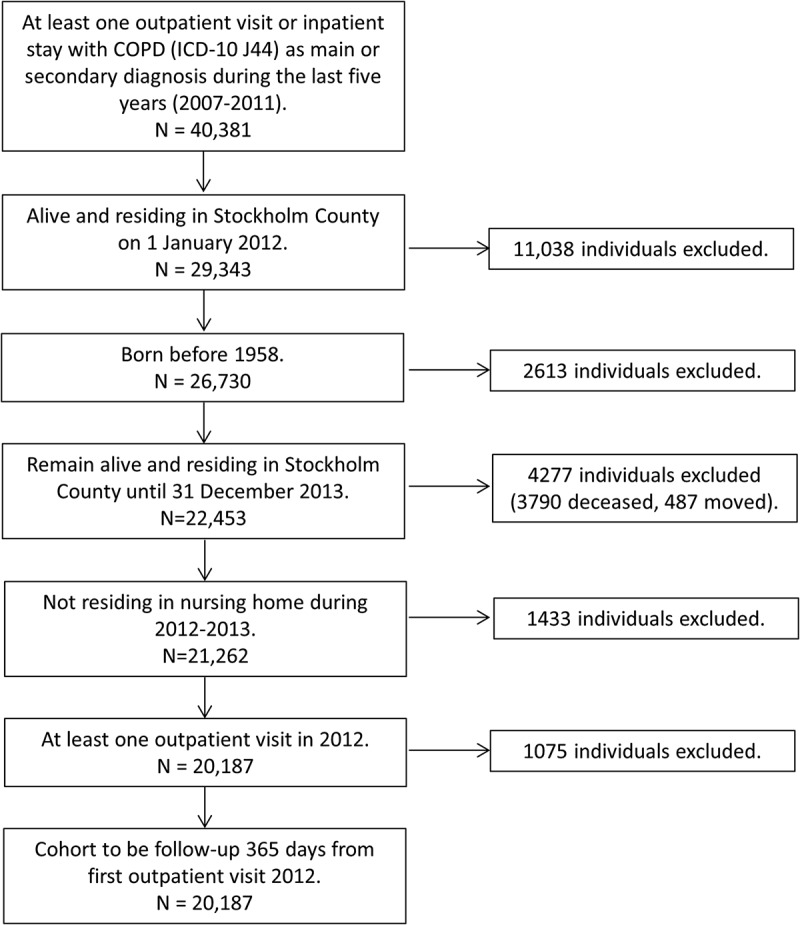
Inclusion of individuals in the study population.​​

We identified 40,381 individuals with at least one main- or secondary diagnosis of COPD (ICD-10 J44, including subcategories) in any type of care (in and outpatient to any caregiver) during 2007–2011. Non-eligible subjects were excluded due to specific exclusion criteria (Figure 1), resulting in a final study population of 20,187 individuals.

### Study design

**Figure 2.**
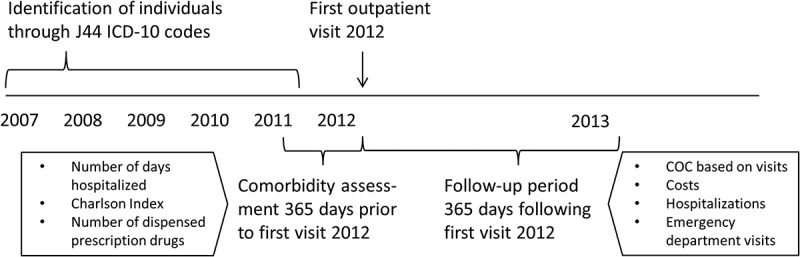
Overview of study design.

In this population based cohort study, modified after Hussey et al. [6], we followed the identified individuals through registries for 365 days after their first outpatient visit (any type) in 2012. Clinically relevant baseline characteristics (age, sex, comorbidity (measured as described below), previous hospitalizations and drug use) were identified using information from the registries 365 days *prior to* this index date (Figure 2). Using data from the follow up period 365 days *after* the index date, we then quantified associations between clinic level COC and three outcome measures: incidence of any hospitalization or emergency department visit, and total costs for health care and pharmaceuticals. In doing this we controlled for age, sex, number of visits and three different comorbidity measures. We refrained from using disease specific outcomes, for example exacerbations defined by ICD-10 codes, due to incomplete diagnosis coding.

### Methods

#### Continuity of care measurement

COC was measured through the Bice–Boxerman COC index. The index was computed using the standard formula (COC=((SUM(nj^2))-n)/(n(n-1)))​​ from j = 1 to j = s) as described by Bice and Boxerman, where *n* is the total number of visits, *nj* is the number of visits to caregiver *j* and *s* is the total number of caregivers.[18] An index of 0 indicates that the individual had different caregivers at all visits (low continuity) and an index of 1 indicates the same caregiver at all visits (high continuity).

We included all visits, including home visits, during the 365-day follow-up period, regardless of diagnosis or provider type. As the role of nurses has been increasingly highlighted in COPD care in recent years, we included not only doctor visits but visits to all kinds of health care professionals. We excluded emergency department visits (as they were already used as outcome), and multiple visits to the same clinic the same day. The index was calculated for 19,704 individuals, using a total of 526,082 individual/day/clinic combinations. A clinic was defined as a center in primary care and as a specific department in hospital care. Individuals with only one visit were not assigned an index.

We finally grouped the individuals into quintiles based on index values. This was motivated by the non-normal distribution of the index, which complicated the interpretation of actual index values.[12]

### Measurement of costs and comorbidity

Costs were calculated based on all outpatient visits, inpatient stays and dispensed prescription drugs for the 365-day follow-up period. For specialized outpatient care and inpatient stays, registry data on diagnosis related group (DRG) costs from the VAL-database was used. DRG is a resource classification system for specialized out-patient and inpatient care. For primary care, home care, emergency care and psychiatry, unit costs were used.^1^ ^1^For individuals with at least one primary health care visit, as well as for individuals enrolled to basic home care, a yearly cost (189/73 Euro (age ≥65/<65) and 393 Euro, respectively) was added to the registered per visit cost. For advanced home care patients, a yearly cost of 811 Euro was added, and per visit costs were estimated to 140 Euro. Visits to local emergency wards were estimated to 116 Euro. Costs for psychiatric care were estimated to 116 Euro (outpatient visit), 231 Euro (emergency department visit) and 578 Euro (inpatient day). Costs were converted to Euros using the average 2012 exchange rate (1 Euro = 8.65 SEK). As the distribution of costs was clearly left skewed, the variable was log transformed prior to regression analysis.

Three comorbidity measures were computed: (1) the number of inpatient days, (2) Charlson index based on all registered diagnoses, and (3) previous drug use, defined as the number of dispensed prescription drugs (unique fifth level, i.e. seven digit or chemical substance, ATC codes). These have previously been found valid indicators of comorbidity [19–21] and were computed based on data during a period 365 days prior to the first visit 2012.

## Statistical analysis

We used regression analysis (SAS software version 9.4 for Windows​​^2^) ^2^Copyright (c) 2015 SAS Institute Inc. SAS and all other SAS Institute Inc. product or service names are registered trademarks or trademarks of SAS Institute Inc., Cary, NC, USA.to quantify associations between COC and the three different outcome measures; logistic regression for hospitalizations and emergency department visits, and multivariate linear regression with log of costs for costs. We controlled for age (discretized into four age groups), sex, number of visits (discretized into nine groups) and comorbidity. The grouping of number of visits was based on correlations with costs – for lower visit volumes the relative cost increase per incremental visit is higher than for higher volumes. To capture this nonlinearity, we created increasingly wide-ranging groups (for example 2–4, 5–9 and 55–99 visits).

To calculate estimates and odds ratios (OR) we used the following reference groups: male individuals, 55–64 years of age, with 2–4 outpatient visits, who had the lowest comorbidity for each of the three indicators, and belonged to the 20% of individuals with highest COC.

For the purpose of sensitivity analysis we fitted four additional models with further (mutually exclusive) exclusion criteria. These were: exclusion of (1) individuals >85 years of age, (2) individuals with <5 visits, (3) individuals with >50 visits, and (4) visits with diagnoses indicating respiratory complications (acute upper respiratory infections (ICD-10 J00–J06), influenza and pneumonia (J09–J18), other acute lower respiratory infections (J20–J22), bronchitis (J40–J42), emphysema (J43), COPD exacerbation (J44.0, J44.1), asthma (J45), bronchiectasis (J47), suppurative and necrotic conditions of lower respiratory tract (J85–J86), respiratory failure (J96.0, J96.1, J96.9), cough (R05) or dyspnea (R06.0)).

Results are presented as mean values ± standard deviations or [95% confidence intervals].

## Ethical considerations

The study was based on anonymized administrative data, which cannot be traced back to identifiable individuals. The analysis of this data is classified as continuous health care quality monitoring. The first author is employed by Stockholm County Council and has solely handled the data processing and analyses in accordance with the county’s regulation about patient confidentiality.

## Results

### Characteristics of the individuals

The study population comprised more women than men, and the most common age group was 65–74 years (Table 1). At baseline, 26% of the individuals had been hospitalized at least once and 73% had dispensed at least seven prescription drugs (23% at least 16) in the last year. During the 365-day follow-up period, almost 70% of the individuals had more than 10 outpatient visits, 40% had at least one emergency department visit and 31% were hospitalized at least once.

**Table 1.** Characteristics of the 20,187 individuals.

	N	%
Baseline characteristics		
Age group		
55–64 years	4665	23
65–74 years	8030	40
75–84 years	5634	28
85+ years	1858	9
Sex		
Male	8341	41
** **Female	11846	59
Comorbidity indicators based on information within 365 days prior to first visit 2012		
Number of days hospitalized		
** **0	14,978	74
** **1–3	2008	10
** **4–7	1191	6
** **8–14	835	4
** **15–29	745	4
** **30–59	326	2
** **60+	104	1
Charlson index		
** **0	7042	35
** **1	7779	39
** **2	2827	14
** **3	1447	7
** **4	578	3
** **5+	514	3
Number of dispensed prescription drugs		
** **0–3	2245	11
** **4–6	3319	16
** **7–10	5047	25
** **11–15	4910	24
** **16–20	2675	13
** **21+	1991	10
Outcomes measured during the follow-up period 365 days from first outpatient visit 2012		
Number of outpatient visits		
** **2–4	2173	11
** **5–9	3988	20
** **10–14	3167	16
** **15–19	2240	11
** **20–29	3071	15
** **30–49	2857	14
** **50–99	1679	8
** **100+	529	3
** **1 (thus excluded from COC measurement)	483	2
Any emergency department visit		
** **No	12,193	60
** **Yes	7994	40
Any hospitalization		
** **No	13,987	69
** **Yes	6200	31

Age group was defined by age at 31 December 2012. COC = continuity of care. Charlson index was calculated based on all registered diagnoses within 365 days prior to first visit 2012. Number of dispensed prescription drugs = unique fifth level ATC codes.

**Figure 3.**
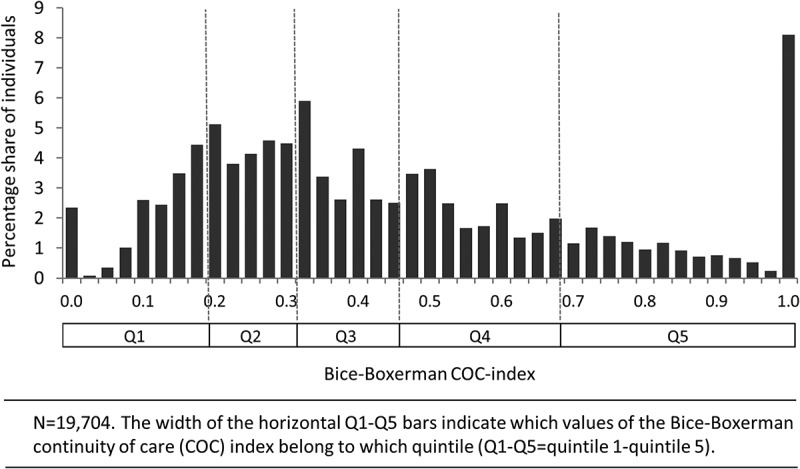
Histogram for Bice–Boxerman continuity of care (COC) index, including quintile grouping in horizontal bars.

Approximately 2% (*N* = 483) of the individuals had <2 visits during the follow-up period, and were hence not assigned a COC index. A total of 1588 individuals (8%) had index values of 1, indicating maximum COC, or all visits to the same clinic. Due to the skewed distribution of the COC-index, the grouping of individuals into quintiles resulted in different width in terms of index values (Figure 3).

Costs were mostly driven by inpatient stays (data not shown), and mean per individual costs were augmented by very high costs for some individuals (7977 ± 15,033 Euro). Mean exponentially back-transformed log of costs was 3647 ± 0.40 Euro per individual, i.e. approaching the median of 3478 Euro.

Median income of the individual’s immediate residential area was initially included as proxy for socioeconomic position, but had no explanatory value in the final model and was hence excluded.

### Descriptive differences between individuals with different COC

Individuals within the 20% of the highest COC (Q5) were on average older than individuals with lower continuity, but had lower incidence of hospitalizations and emergency department visits (Table 2). There was also a clear association between COC and the number of visits. More individuals in Q5 had few (2–4)​​ or many (50–99, 100+) visits whereas none in Q2 had <5 visits. This is partly explained by the mathematical features of the index.^3^ ^3^The Bice–Boxerman COC formula does not result in values in the range 0.2–0.32 (the width of Q2) for any combination of clinics if the number of visits is <5. The only possible values are 0.0, 0.17, 0.33, 0.5 and 1.0 (online Appendix A in Supplementary Material). The level of comorbidity, measured as number of inpatient days, Charlson index, and previous drug use, was more or less similar in all COC quintiles.

**Table 2.** Characteristics of individuals per continuity of care (COC) quintile.

	COC quintile (Q1 = 20% with lowest COC, Q5 = 20% with highest COC)									
	Q1		Q2		Q3		Q4		Q5	
	N	%	N	%	N	%	N	%	N	%
Baseline characteristics										
Age group										
55–64 years	1025	25	846	22	917	23	881	22	810	21
65–74 years	1769	44	1570	41	1583	40	1504	38	1404	36
75–84 years	1026	25	1144	30	1134	29	1136	29	1117	28
85+ years	208	5	285	7	311	8	424	11	610	15
Sex										
Male	1632	41	1477	38	1639	42	1660	42	1698	43
Female	2396	59	2368	62	2306	58	2285	58	2243	57
Comorbidity indicators based on information within 365 days prior to first visit 2012										
Number of days hospitalized										
0	3038	75	2786	72	2940	75	2875	73	2900	74
1–3	445	11	418	11	408	10	364	9	346	9
4–7	232	6	257	7	227	6	233	6	235	6
8–14	154	4	173	4	155	4	185	5	162	4
15–29	115	3	137	4	133	3	183	5	176	4
30–59	38	1	57	1	58	1	89	2	82	2
60+	6	0	17	0	24	1	16	0	40	1
Charlson index										
0	1385	34	1214	32	1353	34	1318	33	1441	37
1	1607	40	1498	39	1505	38	1568	40	1473	37
2	552	14	585	15	559	14	541	14	573	15
3	284	7	309	8	292	7	299	8	259	7
4	92	2	117	3	135	3	119	3	113	3
5+	108	3	122	3	101	3	100	3	82	2
Number of dispensed prescription drugs										
0–3	445	11	302	8	392	10	402	10	517	13
4–6	674	17	513	13	597	15	631	16	764	19
7–10	1032	26	898	23	987	25	1011	26	1021	26
11–15	906	22	1010	26	1040	26	961	24	958	24
16–20	539	13	608	16	528	13	571	14	411	10
21+	432	11	514	13	401	10	369	9	270	7
Outcomes measured during the follow-up period 365 days from first outpatient visit 2012										
Number of outpatient visits										
2–4	610	15	0	0	442	11	282	7	839	21
5–9	925	23	685	18	773	20	916	23	689	17
10–14	728	18	810	21	614	16	608	15	407	10
15–19	507	13	606	16	453	11	385	10	289	7
20–29	640	16	782	20	610	15	556	14	483	12
30–49	418	10	615	16	623	16	624	16	577	15
50–99	186	5	305	8	366	9	412	10	410	10
** **100+	14	0	42	1	64	2	162	4	247	6
Any emergency department visit										
** **No	3439	85	3325	86	3506	89	3485	88	3624	92
** **Yes	589	15	520	14	439	11	460	12	317	8
Any hospitalization										
No	2691	67	2506	65	2698	68	2708	69	2952	75
Yes	1337	33	1339	35	1247	32	1237	31	989	25

### Associations between COC and hospitalizations, emergency department visits and costs

In the main model (where previous drug use was used as comorbidity indicator), the odds ratio for any hospitalization for individuals with low COC (Q1) as compared to high (Q5) was 2.17 [1.95–2.43], and for any emergency department visit 2.06 [1.86–2.28], when controlled for differences in age, sex, number of visits and comorbidity (Table 3).^4^ ^4^Specifications using the other two comorbidity indicators are found in the online appendices (online Appendix B, C in Supplementary Material). The results were consistent also in models where all three comorbidity indicators were included (not shown). Relative increases in total costs amounted to 58%.[52–64] The direction and significance of the results were consistent in the models where we applied further exclusion criteria (data not shown).

**Table 3.** Parameter estimates and odds ratios [95% confidence intervals] for the three outcomes; any hospitalization, any emergency department visit and costs during the follow-up period.

	Odds ratio [95% CI] for any hospitalization as compared to reference group	*p*	Odds ratio [95% CI] for any emergency department visit as compared to reference group	*p*	Relative increase [95% CI] in costs as compared to reference group	*p*
Estimated average for the reference group	0.07 [0.06–0.08]		0.14 [0.12–0.17]		606 [574–641]	
Age group						
55–64 years (ref)						
65–74 years	1.13 [1.04–1.24]	0.01	0.89 [0.82–0.97]	0.01	1.10 [1.06–1.13]	<0.01
75–84 years	1.36 [1.23–1.49]	<0.01	0.99 [0.91–1.08]	0.87	1.08 [1.04–1.11]	<0.01
85+ years	1.88 [1.65–2.13]	<0.01	1.38 [1.22–1.55]	<0.01	1.11 [1.06–1.16]	<0.01
Sex						
Male (ref)						
Female	0.73 [0.68–0.78]	<0.01	0.82 [0.77–0.87]	<0.01	0.89 [0.87–0.91]	<0.01
Number of outpatient visits during the follow-up period						
2–4 (ref)						
5–9	1.76 [1.49–2.09]	<0.01	1.76 [1.53–2.02]	<0.01	1.56 [1.49–1.64]	<0.01
10–14	2.46 [2.07–2.92]	<0.01	2.34 [2.03–2.69]	<0.01	2.29 [2.18–2.41]	<0.01
15–19	3.61 [3.03–4.30]	<0.01	3.40 [2.93–3.94]	<0.01	3.02 [2.87–3.19]	<0.01
20–29	4.33 [3.66–5.13]	<0.01	4.01 [3.48–4.63]	<0.01	3.91 [3.72–4.11]	<0.01
30–49	6.67 [5.63–7.92]	<0.01	5.34 [4.61–6.18]	<0.01	5.50 [5.23–5.80]	<0.01
50–99	11.43 [9.50–13.75]	<0.01	8.51 [7.23–10.03]	<0.01	8.82 [8.31–9.35]	<0.01
100+	19.21 [15.02–24.56]	<0.01	11.75 [9.33–14.80]	<0.01	17.71 [16.27–19.27]	<0.01
Number of dispensed prescription drugs within 365 days prior to first visit 2012						
0–3 (ref)						
4–6	0.93 [0.80–1.07]	0.30	0.97 [0.85–1.10]	0.62	1.26 [1.20–1.32]	<0.01
7–10	0.98 [0.86–1.12]	0.76	0.97 [0.86–1.09]	0.61	1.57 [1.50–1.64]	<0.01
11–15	1.15 [1.01–1.32]	0.04	1.12 [1.00–1.27]	0.06	1.87 [1.79–1.96]	<0.01
16–20	1.36 [1.17–1.57]	<0.01	1.25 [1.10–1.43]	<0.01	2.23 [2.12–2.35]	<0.01
21+	1.75 [1.50–2.05]	<0.01	1.73 [1.50–2.01]	<0.01	2.69 [2.54–2.84]	<0.01
Continuity of care (COC) quintile (Q1 = 20% with lowest COC, Q5 = 20% with highest COC)						
Q5 (ref)						
Q4	1.40 [1.26–1.56]	<0.01	1.41 [1.28–1.56]	<0.01	1.21 [1.17–1.26]	<0.01
Q3	1.57 [1.41–1.75]	<0.01	1.68 [1.52–1.86]	<0.01	1.32 [1.27–1.37]	<0.01
Q2	1.68 [1.50–1.87]	<0.01	1.66 [1.50–1.84]	<0.01	1.41 [1.35–1.46]	<0.01
Q1	2.17 [1.95–2.43]	<0.01	2.06 [1.86–2.28]	<0.01	1.58 [1.52–1.64]	<0.01

**Figure 4.**
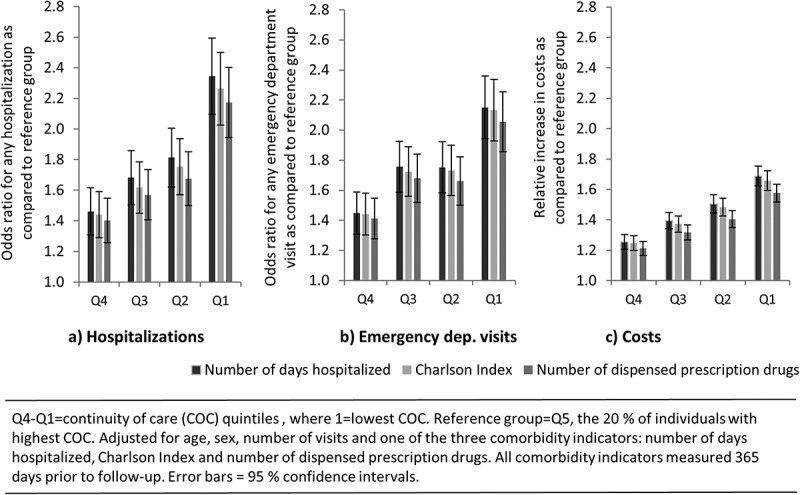
Relative effect of belonging to a lower continuity of care (COC) quintile in relation to the highest quintile (Q5) on (a) any hospitalization, (b) any emergency department visit, (c) costs.​​

We chose the model using previous drug use as comorbidity indicator as the main model for two reasons. First, it had somewhat higher explanatory power for costs (R^2^ = 0.51) than had number of days hospitalized (R^2^ = 0.48) and Charlson index (R^2^ = 0.49), respectively. Second and more importantly, these models provided the most conservative associations between COC and outcomes, as reflected in parameter estimates and odds ratios closer to one (Figure 4).

## Discussion

In all models, individuals belonging to lower COC quintiles had higher probabilities of hospitalizations and emergency department visits, and higher costs. These statistically significant associations followed a proportional pattern; individuals belonging to lower quintiles had worse outcomes than individuals in the next, higher, group. The models explained roughly half of the variation in costs between patients. The findings were stable in the main model as well as in four models with further exclusion criteria. In conclusion, associations between high COC and better outcomes were consistent, relatively strong and followed a logical pattern.

One strength of the study is the full use of a comprehensive dataset of routinely collected data, which enabled individual tracking of patients through all care levels. The inclusion of visits not only to doctors but also to other professions (nurses and physiotherapists, etc.) extended the scope in relation to previous studies, and is in line with modern guidelines for COPD management.[17] Further, the inclusion of all visits, regardless of diagnosis, enabled a more complete picture of the individuals’ health care utilization, regardless of whether they were correctly diagnosed at each time point.

Also, in this study we had access to almost complete DRG cost data for hospitalizations. Although we had to approximate unit costs for other types of care, as costs are largely driven by hospitalizations, we can anticipate our cost estimate to be a fairly valid measure of actual total individual costs. In most previous studies, costs are either not included, or calculated only based on prices or billing data (which discloses cost variability).

Another strength of the study is the use of three different comorbidity measures, yielding consistent results for COC. Model consistency reduces the likelihood that associations are driven by some feature of the comorbidity measure as such, and hence increases the reliability of the study. Among the comorbidity measures, we found previous drug use resulting in somewhat higher explanatory power than other outcomes, which is in accordance with previous studies.[21]

Other authors have suggested that while individuals with higher comorbidity are more likely to have higher costs and worse health care outcomes, they are by definition also more likely to have low continuity as they require care from several different specialists.[7] This potential problem is not supported by our descriptive data, where the level of comorbidity is approximately the same in all COC quintiles.

When interpreting the findings it is important to note that the study population only includes those individuals who remained alive at least 365 days from their first visit in 2012. This inclusion criterion was necessary to make measurement of COC comparable across individuals, but it also has the implication that the results cannot be generalized to the patients with the most severe level of disease.

A limitation of the study is that, although we included several comorbidity measures, we were not able to specifically capture the severity of COPD based on disease specific measures. This was due to data constraints: disease severity is not included in registry data. Potentially, severity could have been indicated through use of specific medications. In a previous study, Make and colleagues [22] showed that COPD maintenance medications are important predictors of exacerbations. Future studies could refine the analysis by stratifying on COPD medication use.

Another limitation is the difficulty to assess the implication of the association between number of visits and COC – with some number of visits even not allowing for all COC levels. As the number of visits is included in the regression models, and the COC quintiles remain significantly associated to outcomes, it is clear that COC harbors an effect that is independent of visit volume, yet it is somewhat difficult to disentangle the two.

A third limitation is that, even though the dose-response-like relationship between COC and outcomes speaks in favor of a causal interpretation, we cannot ascertain that it is COC that *leads* to improved outcomes. This is of importance for the policy implications of the study, and calls for further investigation with other chronic conditions and over several years.

Swedish guidelines on COPD management emphasize inter-professional cooperation, and routinely collected data in Sweden do not distinguish between individual providers. We therefore measured COC at clinic instead of individual clinician level. There are two potential drawbacks to this. First, we cannot ascertain whether the measured associations between higher clinic-level COC and better outcomes is driven at the clinic level as such, or if it is simply an aggregation of a positive effect from high clinician-level COC. Second, although multidisciplinary teams are generally accepted as beneficial for COPD patients, it is not unlikely that some patients experience impaired continuity from visiting different care givers. In effect, although all modern systems for information exchange are in place, from the point of the patient, relational continuity with a particular care provider may be the most important. In order to create a comprehensive understanding of care continuity, a continuity measure such as the one used in this study must therefore be understood in relation to and in combination with the patients’ own experiences.[12,23,24]

## Conclusions

In conclusion, the findings show robust associations between high clinic-level COC, better clinical outcomes and lower health care costs. These results verify the importance of COC, and suggest that clinic-level COC is of relevance to both better outcomes for patients and more efficient use of resources.

## Supplementary Material

Supplementary Material
