# Supplementary material for: Clinic continuity of care, clinical outcomes and direct costs for COPD in Sweden: a population based cohort study
Source: Eur Clin Respir J. 2017 Mar 3;4(1):1290193. doi: 10.1080/20018525.2017.1290193 (PMC5345579; doi:10.1080/20018525.2017.1290193)
Supplement: Supplementary Material [file zecr_a_1290193_sm5017.pdf]

Online Appendix
Clinic continuity of care, clinical outcomes and direct costs for COPD in Sweden: a population based cohort study.
Sveréus, S., Larsson, K. & Rehnberg, C. (*ECRJ,* 2017).

Online appendix A. Possible values for the COC-index when the number of visits is low (example of visit combinations and the resulting COC-index values).

n	j				n(j)^2				Sum (n(j)^2)	((sum(n(j)^2))-n)/(n(n-1))
Total number of visits, all clinics	N.o. visits to clinic A	N.o. visits to clinic B	N.o. visits to clinic C	N.o. visits to clinic D	A^2	B^2	C^2	D^2	Sum A^2:D^2	Bice-Boxerman COC-index
4	1	1	1	1	1	1	1	1	4	0.00
4	4	0	0	0	16	0	0	0	16	1.00
4	2	2	0	0	4	4	0	0	8	0.33
4	1	3	0	0	1	9	0	0	10	0.50
4	2	1	1	0	4	1	1	0	6	0.17
3	1	2	0	0	1	4	0	0	5	0.33
3	3	0	0	0	9	0	0	0	9	1.00
3	1	1	1	0	1	1	1	0	3	0.00
2	2	0	0	0	4	0	0	0	4	1.00
2	1	1	0	0	1	1	0	0	2	0.00

Online Appendix
Clinic continuity of care, clinical outcomes and direct costs for COPD in Sweden: a population based cohort study.
Sveréus, S., Larsson, K. & Rehnberg, C. (*ECRJ,* 2017).

Online appendix B. Parameter estimates and odds ratios [95 % confidence intervals] for the three outcomes, models using Charlson Index as comorbidity indicator.

	Relative increase [95% CI] in costs as compared to reference group	*p*	Odds ratio [95% CI] for any hospitalization as compared to reference group	*p*	Odds ratio [95% CI] for any emergency department visit as compared to reference group	*p*
Estimated average for the reference group	674 [641-709]		0.06 [0.05-0.07]		0.13 [0.11-0.15]	
Age group						
55-64 years (ref)						
65-74 years	1.10 [1.06-1.13]	<0.01	1.10 [1.00-1.20]	0.04	0.88 [0.81-0.95]	<0.01
75-84 years	1.10 [1.06-1.13]	<0.01	1.30 [1.18-1.43]	<0.01	0.97 [0.89-1.06]	0.53
85+ years	1.14 [1.09-1.20]	<0.01	1.80 [1.59-2.04]	<0.01	1.35 [1.20-1.52]	<0.01
Sex						
Male (ref)						
Female	0.97 [0.94-0.99]	0.01	0.80 [0.75-0.85]	<0.01	0.86 [0.81-0.92]	<0.01
Number of outpatient visits during the follow-up period						
2-4 (ref)						
5-9	1.63 [1.55-1.71]	<0.01	1.74 [1.47-2.06]	<0.01	1.75 [1.52-2.01]	<0.01
10-14	2.48 [2.36-2.61]	<0.01	2.43 [2.05-2.88]	<0.01	2.35 [2.04-2.70]	<0.01
15-19	3.39 [3.22-3.58]	<0.01	3.61 [3.03-4.29]	<0.01	3.46 [2.99-4.01]	<0.01
20-29	4.53 [4.31-4.76]	<0.01	4.40 [3.73-5.20]	<0.01	4.17 [3.62-4.80]	<0.01
30-49	6.59 [6.26-6.93]	<0.01	6.87 [5.81-8.12]	<0.01	5.68 [4.92-6.55]	<0.01
50-99	10.99 [10.37-11.65]	<0.01	12.07 [10.07-14.45]	<0.01	9.42 [8.03-11.06]	<0.01
100+	21.20 [19.45-23.10]	<0.01	19.17 [15.01-24.47]	<0.01	12.67 [10.08-15.92]	<0.01
Charlson Index based on all recorded diagnoses within 365 days prior to first visit 2012						
0 (ref)						
1	1.25 [1.21-1.29]	<0.01	1.14 [1.05-1.24]	<0.01	1.12 [1.04-1.20]	<0.01
2	1.46 [1.40-1.51]	<0.01	1.48 [1.34-1.64]	<0.01	1.24 [1.13-1.37]	<0.01
3	1.61 [1.53-1.69]	<0.01	1.71 [1.51-1.94]	<0.01	1.36 [1.20-1.54]	<0.01
4	1.82 [1.69-1.96]	<0.01	2.36 [1.96-2.85]	<0.01	1.71 [1.43-2.06]	<0.01
5+	1.86 [1.72-2.02]	<0.01	2.04 [1.67-2.48]	<0.01	1.56 [1.28-1.89]	<0.01
Continuity of care (COC) quintile (Q1 = 20 % with lowest COC, Q5 = 20 % with highest COC)						
Q5 (ref)						
Q4	1.25 [1.20-1.30]	<0.01	1.44 [1.29-1.61]	<0.01	1.44 [1.30-1.59]	<0.01
Q3	1.37 [1.32-1.43]	<0.01	1.62 [1.45-1.81]	<0.01	1.72 [1.56-1.91]	<0.01
Q2	1.48 [1.42-1.54]	<0.01	1.75 [1.57-1.96]	<0.01	1.73 [1.56-1.92]	<0.01
Q1	1.66 [1.59-1.72]	<0.01	2.26 [2.03-2.53]	<0.01	2.13 [1.93-2.36]	<0.01

Online appendix C. Parameter estimates and odds ratios [95 % confidence intervals] for the three outcomes, models using number of inpatient care days as comorbidity indicator.

	Relative increase [95% CI] in costs as compared to reference group	*p*	Odds ratio [95% CI] for any hospitalization as compared to reference group	*p*	Odds ratio [95% CI] for any emergency department visit as compared to reference group	*p*
Estimated average for the reference group	730 [695-766]		0.06 [0.05-0.07]		0.13 [0.11-0.15]	
Age group						
55-64 years (ref)						
65-74 years	1.12 [1.08-1.16]	<0.01	1.13 [1.03-1.24]	0.01	0.88 [0.82-0.96]	<0.01
75-84 years	1.12 [1.08-1.16]	<0.01	1.33 [1.21-1.46]	<0.01	0.97 [0.89-1.06]	0.55
85+ years	1.14 [1.09-1.20]	<0.01	1.76 [1.55-2.00]	<0.01	1.31 [1.16-1.47]	<0.01
Sex						
Male (ref)						
Female	0.94 [0.92-0.97]	<0.01	0.77 [0.72-0.82]	<0.01	0.86 [0.80-0.91]	<0.01
Number of outpatient visits during the follow-up period						
2-4 (ref)						
5-9	1.65 [1.57-1.72]	<0.01	1.72 [1.45-2.04]	<0.01	1.73 [1.50-1.98]	<0.01
10-14	2.54 [2.42-2.67]	<0.01	2.42 [2.04-2.87]	<0.01	2.31 [2.01-2.67]	<0.01
15-19	3.51 [3.33-3.70]	<0.01	3.60 [3.02-4.28]	<0.01	3.40 [2.94-3.94]	<0.01
20-29	4.66 [4.43-4.89]	<0.01	4.33 [3.66-5.11]	<0.01	4.04 [3.51-4.65]	<0.01
30-49	6.68 [6.35-7.03]	<0.01	6.52 [5.51-7.71]	<0.01	5.34 [4.63-6.15]	<0.01
50-99	10.79 [10.18-11.44]	<0.01	10.83 [9.03-12.98]	<0.01	8.53 [7.27-10.01]	<0.01
100+	20.03 [18.38-21.83]	<0.01	16.41 [12.82-21.01]	<0.01	11.20 [8.90-14.10]	<0.01
Number of inpatient care days within 365 days prior to first visit 2012						
0 (ref)						
1-3	1.26 [1.21-1.31]	<0.01	1.60 [1.44-1.77]	<0.01	1.55 [1.40-1.71]	<0.01
4-7	1.45 [1.38-1.53]	<0.01	2.11 [1.86-2.40]	<0.01	1.82 [1.60-2.06]	<0.01
8-14	1.64 [1.54-1.75]	<0.01	2.59 [2.23-3.02]	<0.01	2.05 [1.76-2.38]	<0.01
15-29	1.76 [1.65-1.88]	<0.01	2.79 [2.37-3.28]	<0.01	1.95 [1.66-2.30]	<0.01
30-59	2.04 [1.85-2.25]	<0.01	3.36 [2.61-4.31]	<0.01	1.97 [1.54-2.51]	<0.01
60+	2.69 [2.27-3.19]	<0.01	4.54 [2.82-7.31]	<0.01	1.65 [1.08-2.52]	0.02
Continuity of care (COC) quintile (Q1 = 20 % with lowest COC, Q5 = 20 % with highest COC)						
Q5 (ref)						
Q4	1.25 [1.21-1.30]	<0.01	1.46 [1.31-1.63]	<0.01	1.45 [1.31-1.60]	<0.01
Q3	1.39 [1.34-1.45]	<0.01	1.68 [1.50-1.88]	<0.01	1.76 [1.59-1.94]	<0.01
Q2	1.51 [1.45-1.57]	<0.01	1.81 [1.62-2.03]	<0.01	1.75 [1.58-1.94]	<0.01
Q1	1.69 [1.62-1.76]	<0.01	2.34 [2.10-2.62]	<0.01	2.15 [1.94-2.38]	<0.01
